# Whole brain radiotherapy (WBRT) for leptomeningeal metastasis from NSCLC in the era of targeted therapy: a retrospective study

**DOI:** 10.1186/s13014-020-01627-y

**Published:** 2020-07-31

**Authors:** Junjie Zhen, Lei Wen, Mingyao Lai, Zhaoming Zhou, Changguo Shan, Shaoqun Li, Tao Lin, Jie Wu, Wensheng Wang, Shaoqiang Xu, Da Liu, Ming Lu, Dan Zhu, Longhua Chen, Linbo Cai, Cheng Zhou

**Affiliations:** 1 Department of Radiation Oncology, Nanfang Hospital, Southern Medical University, Guangzhou, 510515 P. R. China; 2 Department of Oncology, Guangdong Sanjiu Brain Hospital, Guangzhou, 510510 P. R. China; 3 Department of Radiation Medicine, School of Public Health, Southern Medical University, Guangzhou, China; 4 Department of Neurosurgery, Guangdong Sanjiu Brain Hospital, Guangzhou, China; 5 Department of Radiology, Guangdong Sanjiu Brain Hospital, Guangzhou, China; 6 Department of Laboratory Medicine, Guangdong Sanjiu Brain Hospital, Guangzhou, China; 7 Translational Radiation Oncology, German Cancer Consortium (DKTK), National Center for Tumor Diseases (NCT), German Cancer Research Center (DKFZ), Heidelberg, Germany

**Keywords:** Leptomeningeal metastasis, Whole brain radiotherapy (WBRT), Non-small cell lung cancer (NSCLC), EGFR mutation, Prognosis

## Abstract

**Background and purpose:**

Leptomeningeal metastasis (LM) is a rare but detrimental complication in patients with non-small cell lung cancer (NSCLC). Although whole brain radiotherapy (WBRT) is used to eliminating cancer cells or microscopic foci, it is becoming less favorable due to the concerns over neurocognitive toxicity. This study aimed to re-evaluate the role of WBRT in the setting of modern targeted therapy.

**Materials and methods:**

From December 2014 to March 2019, 80 NSCLC patients with cytologically and/or radiologically proven LM diagnosis were retrospectively analyzed.

**Results:**

The median OS (mOS) after diagnosis of LM was 8.0 (95%CI: 4.4 to 11.6) months, and the one-year OS was 39.4%. The mOS for EGFR-mutated LM patients was 12.6 (3.0 to 22.2) months versus only 4.1 (2.8 to 5.4) for patients with wild-type EGFR (*P* < 0.001). Younger patients (< 53.5 yrs.) appeared to have a better OS than older patients (≥53.5 yrs.) (12.6 vs. 6.1, *P* = 0.041). No survival benefits were found in EGFR-mutated patients who received WBRT (*P* = 0.490). In contrast, mOS was significantly prolonged in wild-type EGFR patients with WBRT versus non-WBRT (mOS: 8.0 vs. 2.1, *P* = 0.002). Multivariate analysis indicated that WBRT (*P* = 0.025) and younger age (*P* = 0.048) were independent prognostic factors that predicted prolonged survival for wild-type EGFR LM patients from NSCLC.

**Conclusion:**

Our study demonstrated that WBRT has clear survival advantages for patients with wild-type EGFR, and molecular biological stratification of LM patients for WBRT is highly recommended.

## Introduction

Leptomeningeal metastasis (LM) is a deleterious complication that occurs in 3–5% of patients with advanced non-small cell lung cancer (NSCLC) [1]. In recent years, the growing incidence of LM is likely due to improved supportive care as well as prolonged survival in patients with targetable mutations, particularly epidermal growth factor receptor (EGFR) and anaplastic lymphoma kinase (ALK) mutations [2, 3]. Nevertheless, LM is associated with a very poor prognosis [4–6].

The last decade saw considerably changes in management of NSCLC by appreciating the molecular characterization of the disease. These findings have led to biological stratifications of the patients as well as the discovery of a variety of targeted cancer drugs. For instance, the sequential development of tyrosine kinase inhibitors (TKIs) as well as check point inhibitors for program death ligand 1 (PD-L1) were enabled [7, 8]. As a result, the prognosis and quality of life for NSCLC patients has improved. Unfortunately, this is not the case in LM. Many clinical trials in NSCLC excluded patients with LM. There have been a few retrospective studies evaluating the prognosis of LM from NSCLC in the EGFR mutation subgroup. The median overall survival of these studies varies from 3 to 4 months [9, 10] to 9–12 months [11, 12].

Local-regional treatments including whole brain radiotherapy (WBRT), intrathecal chemotherapy (ITC), and ventriculoperitoneal (VP) shunt operations play a critical role in relieving neurological symptoms caused by intracranial hypertension in LM [13–15]. Although non-invasive whole brain radiotherapy (WBRT) seems to be effective in palliating neurologic signs and symptoms [13], WBRT has not been widely accepted for treating LM due to its plausible amelioration in survivals at the expense of potentially neurocognitive toxicity [11, 16]. Several important questions have yet to be answered: 1) whether WBRT has an impact on survival outcomes; 2) if so, which sub-population will benefit from WBRT; 3) what are the optimal doses of WBRT for patients with different clinical and biological backgrounds. Herein, we aimed to understand the role of WBRT in treatment of LM from NSCLC based on a retrospective cohort in China. We further evaluated the clinicopathological factors and survival of LM patients who underwent various local or systemic treatments in the era of molecular targeted therapy.

## Materials and methods

### Patients

Patients diagnosed with LM from NSCLC at our institution were retrospectively analyzed from December 2014 to March 2019. The inclusion criteria included: (1) definitive cases with confirmed malignant cells in CSF, and/or (2) diffuse linear leptomeningeal enhancements following the gyri, sulci or ependymal surface by gadolinium-enhanced magnetic resonance imaging (MRI) scan, and (3) gene tests of EGFR mutation. We excluded patients with ALK translocation (*n* = 5) and those whose EGFR mutation status was unknown (*n* = 20). Data on the clinical characteristics of patients as well as disease-related features including radiology, histology, molecular status of EGFR mutation, and treatment regimens were reviewed. This study was approved by the Ethics Committee of Guangdong Sanjiu Brain Hospital.

### Treatments

Systemic therapy for patients with LM was administered according to NCCN guidelines for NSCLC including EGFR tyrosine kinase inhibitors (TKIs) for those with EGFR mutations and chemotherapy with or without bevacizumab. WBRT was implemented with either 30 Gy in 15 fractions of 2 Gy, or 36 Gy in 18 fractions of 2 Gy, delivered 5 days a week, by intensity-modulated radiation therapy (IMRT) or 3-dimensional conformal radiation therapy (3D-CRT). Five patients with concurrent brain metastasis were treated by stereotactic radiosurgery (SRS) or fractioned SRS (12–24 Gy/ 1–3 fractions) using the Novalis Tx® system. For patients with significant clinical symptoms of meningeal irritation, surgical interventions including ventriculoperitoneal (VP) shunt, external ventricular drainage (EVD), or lumbar cistern drainage (LCD) were provided especially for those with elevated intracranial pressure prior to radiotherapy. Adverse events were graded according to the National Cancer Institute Common Toxicity Criteria for Adverse Events version 4.0. Adverse events were graded according to the National Cancer Institute Common Toxicity Criteria for Adverse Events version 4.0.

### Statistics

The primary endpoint of our study was median overall survival (OS) determined as the time from initial diagnosis of LM to death from any cause or censored at the date of last follow-up unless otherwise specified. The radiological signs of LM are difficult for assessment; therefore, the progression free survival and overall response rates were not calculated in this retrospective cohort. Continuous data were presented as the median (minimum-maximum). Categorical data were presented as quantities and proportions. The overall survival was estimated using the Kaplan-Meier product-limited method with ‘survival’ package [17] in R software; survival curves were compared between groups using a log-rank test. Univariable and multivariable Cox proportional hazards regression models were performed to evaluate the influence of the clinical and pathologic parameters on mortality of NSCLC patients with LM. Continuous data such as age and Karnofsky Performance Status (KPS) score were divided into two subgroups via a cut-off value using the median value of the cohort for univariable and multivariable regression modeling. Statistical analyses used R software version 3.5.1. All statistical assessments were two-sided, and *P* < 0.05 was considered to be statistically significant.

## Results

### Patient characteristics

Eighty NSCLC patients with LM were eligible for our study and consisted of 44 males and 36 females. Baseline characteristics of all included patients are listed below (Table 1). The median age at diagnosis of LM was 53.5 (range: 30 to 78 years). Most patients had adenocarcinoma histology (73/80, 91.3%). Malignant cells were found in 55 patients from CSF while the remaining 25 patients were diagnosed with LM according to magnetic resonance imaging (MRI) findings and clinical presentations. Nineteen patients had synchronous LM when diagnosed with NSCLC while 61 patients progressed to LM after at least one line of systematic therapy. The median time from diagnosis of NSCLC to metachronous LM was17.6 month (range: 3.0 to 73.0). Forty-two (52.5%) patients had brain metastasis when diagnosed with LM. Fifty-one (63.8%) patients also had extra-central nervous system (CNS) metastasis mainly involving lung, liver, and lymph nodes (Table 2).

**Table 1:** Patient characteristics of 80 NSCLC patients with LM

Variable	N (%)
**Total**	80 (100.0)
**Gender**	
Male	44 (54.1)
Female	36 (45.9)
**Age, years**	
Median (range)	53.5 (30–78)
**Histology**	
Adenocarcinoma	73 (91.3)
Squamous	7 (8.8)
**Initial stage**	
I-III	25 (31.3)
IV	55 (68.8)
**Previous lines of systematic therapy**	
0	19 (23.8)
1	29 (36.3)
2	19 (23.8)
≥ 3	13 (16.3)
**Previous EGFR TKI prior to LM**	46 (57.5)
**EGFR mutation**	
EGFR 19del	25 (31.3)
EGFR L858R	18 (22.5)
EGFR T790M	5 (6.3)
EGFR 20INS	1 (1.3)
Wild-type	31 (38.8)

*Abbreviations*: *EGFR* Epidermal growth factor receptor, *TKI* Tyrosine kinase inhibitors, *LM* Leptomeningeal metastasis

**Table 2:** Clinical presentation and treatment of 80 NSCLC patients with LM

Variable	N (%)
**Timing of metastasis**	
Synchronous	19 (23.8)
Metachronous	61 (76.3)
**Time from diagnosis of lung cancer to LM, months**	
Median (range)	12.5 (−0.2 to 73.0)
**KPS score**	
≥ 80	21 (26.3%)
70–50	41 (51.3%)
≤ 40	18 (22.5%)
**GCS score**	
Less than 15	16 (20.0)
**With CNS symptoms**	80 (100.0)
**Modality of LM diagnosis**	
MRI	25 (31.3)
CSF cytology	4 (5.0)
MRI + CSF cytology	51 (63.8)
**Concurrent brain metastasis**	42 (52.5)
**Extra-CNS metastasis**	51 (63.8)
**Local treatment for LM**	
WBRT	38 (47.5)
VP shunt	6 (7.5)
Ventricular external drainage	7 (8.8)
Lumbar cistern drainage	3 (3.8)
**Systematic treatment for LM**	
Osimertinib	25 (31.3)
Gefitinib	12 (15.0)
Erlotinib	4 (5.0)
Icotinib	5 (6.3)
Afatinib	1 (1.3)
Gefitinib followed by Osimertinib	2 (2.5)
Bevacizumab	9 (11.3)
Chemotherapy	36 (45.0)

*Abbreviations*: *CNS* Central nervous system, *CSF* Cerebrospinal fluid, *MRI* Magnetic resonance imaging, *VP* Ventriculoperitoneal, *KPS* Karnofsky performance status, *GCS* Glasgow coma scale, *EGFR* Epidermal growth factor receptor, *TKI* Tyrosine kinase inhibitors, *LM* Leptomeningeal metastasis, *WBRT* Whole brain radiotherapy, *Gy* Gray, *HR* Hazard ratio, *CI* Confidence interval

EGFR mutations were determined by gene panel sequencing or polymerase chain reaction (PCR)-based assays on different samples (37 from tumor tissue, 24 from CSF, 9 from both CSF and serum, 6 from serum, 3 from both tumor tissue and serum, and 1 from pleural effusion). Of these, 25 patients had EGFR exon 19 deletion, 18 had EGFR exon L858R mutation, and 1 patient had EGFR exon 20 insertion. The T790M mutations were reported in five patients. The remaining 31 patients were wild-type EGFR. Forty-six (57.5%) patients had received EGFR TKI therapy prior to the diagnosis of LM (Table 1).

### Treatment after diagnosis of LM

Thirty-eight (47.5%) patients received WBRT out of a total of 30–36 Gy/15–18 fractions. Stereotactic radiosurgery was implemented in 5 patients for the treatment of concurrent brain metastasis. Sixteen patients underwent surgical interventions including 6 VP shunts, 7 external ventricle drainage cases, and 3 lumbar cistern drainages. With respect to systematic therapy after diagnosis of LM, 25 patients received osimertinib, 22 patients received first- or second-generation EGFR TKI (including gefitinib in 12 patients, erlotinib in 4, icotinib in 5, and afatinib in 1 patients). Two patients received gefitinib followed by osimertinib; 36 patients were given platinum-based chemotherapy, and 9 of them were supplemented with bevacizumab (Table 2).

### Survival analysis

**Fig. 1:**
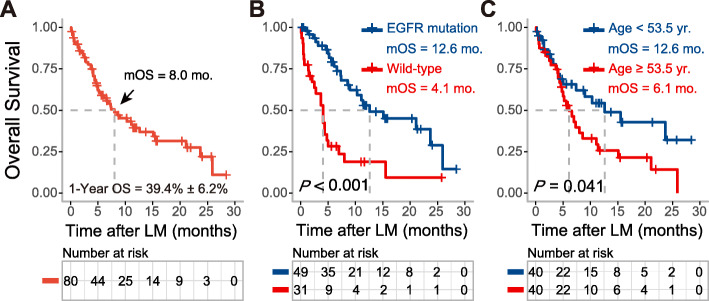
Overall survival (OS) of leptomeningeal metastasis (LM) from NSCLC cohort and Kaplan-Meier estimates based on clinical characteristics. **a** The median OS of all the patients after diagnosis of LM was 8.0 months; **b** The OS for LM patients with EGFR mutation was significantly improved than those wild-type EGFR (*P* < 0.001); **c** The OS for LM patients aged < 53.5 years was significantly extended than those ≥53.5 years (*P* = 0.041)

The median follow-up time was 8.6 months (range: 1 to 28.4 months); 47 patients died within this period, 29 patients were still alive, and 4 were lost to follow-up. The median OS after diagnosis of LM was 8.0 months (95% confidence interval [CI]: 4.4 to 11.6 months), and one-year OS was 39.4% (Fig. 1a). Subgroup analysis revealed that the median OS for EGFR-mutated patients was 12.6 months (95% CI: 3.0 to 22.2 months), which was significantly longer than those with wild-type EGFR LM patients (4.1 months, 95% CI: 2.8 to 5.4 months, *P* < 0.001) (Fig. 1b). Patients who received EGFR TKI were identified to have a better OS than non-TKI treatment (11.1 vs. 2.5 months, *P* < 0.001).

We next asked whether age at diagnosis of LM has an impact on survival and prognosis; subgroup analysis indicated that younger age (< 53.5 yrs.) appeared to gain a favorable OS versus older ones (≥53.5 yrs.) (12.6 vs. 6.1 months, *P* = 0.041) (Fig. 1c). No significant difference was found in OS with reference to sex, KPS score, Glasgow Coma Scale (GCS) score, concurrent brain metastasis, chemotherapy, and bevacizumab therapy (Table 3). Multivariate analysis showed that EGFR mutations (*P* = 0.013, HR 0.39, 95% CI: 0.186 to 0.820) and younger age (*P* = 0.025, HR 0.492, 95% CI: 0.265 to 0.915) were still independent prognostic factors that predicted better survival (Table 3).

**Table 3:** Univariate and multivariate analysis for survival in 80 NSCLC patients with LM

	Univariate analysis		Multivariate analysis	
	HR (95%CI)	***P***	HR (95%CI)	***P***
**Gender (female vs. male)**	0.950 (0.531 to 1.698)	0.862		
**Age (< 53.5 vs. ≥53.5)**	0.545 (0.301 to 0.987)	0.043	0.492 (0.265 to 0.915)	0.025
**KPS (< 80 vs. ≥80)**	1.298 (0.640 to 2.632)	0.470		
**GCS (15 vs. ≤14)**	1.126 (0.572 to 2.220)	0.731		
**Concurrent brain metastasis (yes vs. no)**	1.039 (0.585 to 1.846)	0.895		
**EGFR mutation (yes vs. no)**	0.288 (0.159 to 0.522)	0.000	0.390 (0.186 to 0.820)	0.013
**EGFR TKI after LM (yes vs. no)**	0.260 (0.138 to 0.487)	0.000	0.549 (0.491 to 1.039)	0.053
**WBRT (yes vs. no)**	0.565 (0.315 to 1.013)	0.053	0.697 (0.373 to 1.306)	0.260
**Chemotherapy (yes vs. no)**	0.873 (0.480 to 1.589)	0.657		
**Bevacizumab (yes vs. no)**	1.909 (0.677 to 5.386)	0.222		

*Abbreviations*: *NSCLC* non-small-cell lung cancer, *KPS* Karnofsky Performance Status, *GCS* Glasgow Coma Scale, *EGFR* epidermal growth factor receptor, *TKI* tyrosine kinase inhibitors, *LM* leptomeningeal metastasis, *WBRT* whole brain radiotherapy, *Gy* gray, *HR* hazard ratio, *CI* confidence interval

### Distinct survival impacts of WBRT

**Fig. 2:**
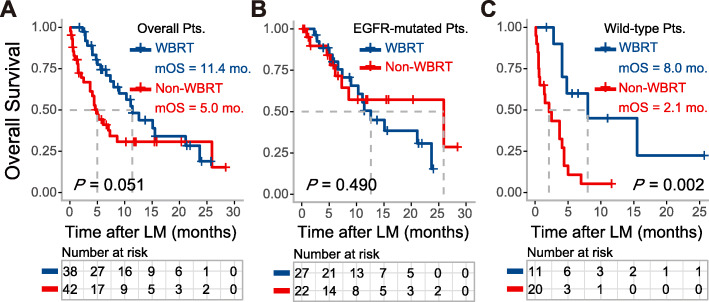
Kaplan-Meier estimates for LM patients from NSCLC with or without WBRT. **a** The comparison of OS between overall patients with or without WBRT (*P* = 0.051); **b** The comparison of OS between EGFR-mutated patients with or without WBRT (*P* = 0.490); **c** The comparison of OS between wild-type EGFR patients with or without WBRT (*P* = 0.002)

To study the therapeutic effects of WBRT, the OS of patients who received WBRT (WBRT group, *n* = 38) were evaluated versus those without WBRT (non-WBRT group, *n* = 42). Although no significant difference in OS was found between two arms (*P* = 0.051), the median OS of WBRT group was numerically doubled compared to non-WBRT group (11.4 vs. 5.0 months). (Fig. 2a). We next explored the consequences of WBRT in LM patients with different mutation backgrounds via sub-group analysis. For patients harboring EGFR mutations, no survival benefits were observed with WBRT treatment by univariate analysis (WBRT vs. non-WBRT: *P* = 0.49, HR 0.73, 95%CI 0.297 to 1.794) (Fig. 2b). Interestingly, with respect to patients with wild-type EGFR, the survival advantages of WBRT was exceptionally established (median OS: 8.0 vs. 2.1 months, *P* = 0.002, HR 0.229, 95%CI 0.083 to 0.632) (Fig. 2c). Multivariate analysis consistently showed that WBRT (*P* = 0.025) and younger age (*P* = 0.048) were independent prognostic factors that predicted favorable overall survival in patients with wild-type EGFR (Table 4).

**Table 4:** Univariate and multivariate analysis for survival in 31 LM patients with wild-type EGFR

	Univariate analysis		Multivariate analysis	
	HR (95%CI)	***P***	HR (95%CI)	***P***
**Gender (female vs. male)**	0.777 (0.328 to 1.844)	0.567		
**Age (< 53.5 vs. ≥53.5)**	0.397 (0.153 to 1.029)	0.057	0.353 (0.125 to 0.993)	0.048
**KPS (< 80 vs. ≥80)**	0.654 (0.233 to 1.831)	0.419		
**GCS (15 vs. ≤ 14)**	0.687 (0.232 to 2.033)	0.497		
**Concurrent brain metastasis (yes vs. no)**	0.891 (0.380 to 2.088)	0.791		
**WBRT (yes vs. no)**	0.229 (0.083 to 0.632)	0.004	0.300 (0.105 to 0.858)	0.025
**Chemotherapy (yes vs. no)**	0.422 (0.176 to 1.014)	0.054	0.487 (0.187 to 1.268)	0.140
**Bevacizumab (yes vs. no)**	0.034 (0.000 to 23.167)	0.310		

*Abbreviations*: *KPS* Karnofsky performance status, *GCS* Glasgow coma scale, *EGFR* Epidermal growth factor receptor, *WBRT* Whole brain radiotherapy, *Gy* Gray, *HR* Hazard ratio, *CI* Confidence interval

### Toxicity of WBRT

Two patients in WBRT group suspended irradiation due to worsening headache, both of them completed irradiation after decompression surgery were conducted. Other WBRT related toxicities including grade 1 to 2 nausea/vomiting in 7 patients, grade 1 transient headache in 5 patients, grade 1 radiation dermatitis in 3 patients and grade 1 hearing impaired in 1 patient.

## Discussion

This cohort study shows a relatively long median OS (8.0 months) in patients diagnosed with LM from NSCLC in a single institution, particularly in an EGFR-mutated group (12.6 months) versus previous published data ranging from 3 to 6 months [4–6, 9, 10]. The most important factor in NSCLC in recent years is an improved understanding of molecular characteristics that leads to precision therapy of metastatic NSCLC [18]. Nonetheless, high-level evidence of targeted therapy for the treatment of LM from NSCLC has been scarce since the Iressa Pan-Asia Study (IPASS) [18] and most subsequent studies have excluded patients with LM from clinical trials. A large-scale Chinese study of 5387 lung cancer patients found that EGFR-mutated subjects have a significantly higher risk for LM versus wild-type subjects (9.4% vs. 1.7%). The OS after LM was remarkably improved in the TKI therapy group versus non-TKI treatment (10.0 vs. 3.3 months) [2]. Another multicenter retrospective analysis from Europe consisting of 92 EGFR-mutated NSCLC patients with LM showed that the median OS from diagnosis of LM was 6.1 months; re-challenging with TKI in TKI-failed patients showed a better prognosis versus patients without further therapy (7.6 vs. 4.2 months) [19].

Besides evidence from retrospective studies, several preliminary prospective studies explored the efficiency of targeted therapy for EGFR-mutated LM patients. Nanjo et al. [20] examined the effects of the third-generation EGFR TKI osimertinib in a prospective pilot study including 13 patients with T790M-positive NSCLC LM patients after failure of first- or second-line EGFR TKI. Six out of eight patients achieved CNS improvement, and extra-CNS improvement was seen in five patients. The median progression-free survival for all 13 patients was 7.2 months. The BLOOM study is a phase I clinical trial to assess the safety and activity of AZD3759 or AZD9291 in patients with EGFR-mutated advanced-stage NSCLC with central nervous system metastasis. AZD3759 [21] showed a tolerable safety profile at a dose of 200 mg twice daily. Of 18 patients with LM pretreated with EGFR TKI, five (28%) patients had a confirmed response, and 14 (78%) achieved confirmed disease control. AZD9291 [22] also showed a manageable safety profile with the investigator-assessed median PFS 8.6 months and median OS 11.0 months in EGFR-mutant NSCLC with LM.

There is also limited progress with respect to wild-type EGFR NSCLC patients with LM. WBRT is an important choice of local treatment in LM patients by eradicating cancer cells or microscopic foci at distant sites within the brain [13]. Owning to the neurologic toxicity, the role of WBRT has long been a controversial issue. There is still no consensus on whether WBRT could improve survival for patients with LM from NSCLC [16]. Su*et al.* showed that patients who received WBRT had a longer overall survival versus those who did not receive WBRT; WBRT was an independent favorable factor that predicted better survival [9]. Liao et al. reported that WBRT prolonged median OS from 2.4 months to 10.9 months in patients with LM from NSCLC [23]. However, a study from the US suggested that survival was not improved in 56 patients who received WBRT versus 69 patients who received no WBRT [24]. A multicenter study from 11 Dutch hospitals reported that median survival of NSCLC patients with LM was only 3.1 months, and WBRT did not affect survival after LM diagnosis based solely on EGFR-mutant NSCLC LM patients [25].

A probable reason for this discrepancy between the survival impacts of WBRT may be a mixture of patients with a diversity of clinical and molecular biological background. In fact, the therapeutic response to WBRT varies with the number of factors including the Eastern Cooperative Oncology Group (ECOG) performance status (PS) 1, time to leptomeningeal metastasis following NSCLC diagnosis, as well as lack of brain metastasis [26]. Our study reported a doubling of OS with statistical marginal difference between patients who received WBRT and the non-WBRT group (11.4 vs. 5.0 months, *P* = 0.051). Our subgroup analysis reported that 49 patients with definite EGFR mutation gained almost no benefit from WBRT. This agrees with Yan et al. who found that WBRT did not improve the overall survival of EGFR-mutated patients with LM [11]. We further reported that the overall survival in wild type patients with LM is noticeably enhanced. Su et al. showed that WBRT improved the overall survival in patients mostly (91.3%) had wild-type EGFR or unknown status [9]. As a result, LM patients with wild-type EGFR favor WBRT but EGFR-mutated patients do not — this is a key factor that might explain the various mOS reported from different studies.

Our study further implicated younger age (< 53.5 years) as an independent prognostic factor that predicts better survival. Younger patients tend to have fewer comorbidities and more tolerance to toxicities induced by a variety of treatments. LM commonly presents with increased intracranial hypertension-associated symptoms such as consistent headache, nausea, vomiting, and even disturbed consciousness. These signs may not be fundamentally alleviated by dehydration treatment alone. Surgical intervention such as ventriculoperitoneal shunt (VP shunt) plays an important role in these intractable situations. VP shunting could offer an effective palliative option for symptom relief of severe headache and improved quality of life in LM patients [14, 27]. Intrathecal chemotherapy could be implemented repeatedly and safely via implantation of an ommaya reservoir [28]. In our cohort, a small proportion of patients (16/80, 20%) had undergone surgical interventions including VP shunt, VED, and LCD. Those surgical interventions offer a supportive role for WBRT (*data not shown*).

Immunotherapy, particularly inhibitors of the programmed death-1 (PD-1)/PD-ligand 1 (PD-L1) pathways—have a greatly modified NSCLC treatment [29]. However, evidence of immunotherapy for the treatment of LM is limited because the tight junctions between ependymal cells in the choroid plexus are less permeable to T cells or anti-PD-1 monoclonal antibodies reaching the leptomeninges and CSF [30]. Few data are available except several case reports on the activity of PD-1/PD-L1 inhibitors for the treatment of LM. Kamath et al. reported a radiologically stable and neurologically intact treatment that lasts for at least 20 months in a woman with reginal bulky LM from NSCLC by combined treatment of stereotactic radiosurgery and pembrolizumab [31]. Gionet al. also reported neurological improvement after nivolumab treatment in a patient with NSCLC and symptomatic LM. The activity of pembrolizumab in LM was also investigated in a phase II study (NCT03091478) [32].

Among potential limitations of this study could be the relatively small number of patients for the subgroup analysis that might reduce the quality of the conclusions. Periodic follow-up on quality of life was also absent in this cohort.

In conclusion, this study revealed that the median overall survival in our cohort is higher than histological experience. EGFR mutations were identified as a prognostic factor that predicts favorable survival in NSCLC patients with LM. Our study also showed that WBRT could significantly improve the survival outcome of LM patients with wild-type EGFR. However, LM patients with EGFR mutations are likely to gain limited benefits from WBRT. Molecular biological stratifications of LM patients for WBRT are therefore recommended for routine clinical practice. Further clinical and mechanistic investigations for optimal radiation dose-fractionation regimens or the development of combined radio-sensitive agents are highly warranted.

## Data Availability

The datasets used and/or analyzed during the current study are available from the corresponding author on reasonable request.

## Abbreviations

ALK: Anaplastic lymphoma kinase
CI: Confidence interval
CNS: Central nervous system
CSF: Cerebrospinal fluid
ECOG: Eastern Cooperative Oncology Group
EGFR: Epidermal growth factor receptor
EVD: External ventricular drainage
GCS: Glasgow Coma Scale
Gy: Gray
HR: Hazard ratio
IMRT: Intensity modulated radiation therapy
ITC: Intrathecal chemotherapy
KPS: Karnofsky Performance Status
LCD: Lumbar cistern drainage
LM: Leptomeningeal metastasis
MRI: Magnetic resonance imaging
NSCLC: Non-small-cell lung cancer
OS: Overall survival
PCR: Polymerase chain reaction
PS: Performance status
PD-1: Program death 1
PD-L1: Program death ligand 1
TKI: Tyrosine kinase inhibitors
VP: Ventriculoperitoneal
WBRT: Whole brain radiotherapy
3D-CRT: 3-dimensional conformal radiation therapy
